# Inspiratory resistances facilitate the diaphragm response to transcranial stimulation in humans

**DOI:** 10.1186/1472-6793-6-7

**Published:** 2006-07-29

**Authors:** Chrystèle Locher, Mathieu Raux, Marie-Noelle Fiamma, Capucine Morélot-Panzini, Marc Zelter, Jean-Philippe Derenne, Thomas Similowski, Christian Straus

**Affiliations:** 1 Université Pierre et Marie Curie-Paris 6, UPRES EA 2397, Paris, France; 2 Service de Pneumologie, Centre Hospitalier de Meaux, Meaux, France; 3 Assistance Publique-Hôpitaux de Paris, Service de Pneumologie, Groupe Hospitalier Pitié-Salpêtrière, Paris, France; 4 Assistance Publique-Hôpitaux de Paris, Service Central d'Explorations Fonctionnelles Respiratoires, Groupe Hospitalier Pitié-Salpêtrière, Paris, France

## Abstract

**Background:**

Breathing in humans is dually controlled for metabolic (brainstem commands) and behavioral purposes (suprapontine commands) with reciprocal modulation through spinal integration. Whereas the ventilatory response to chemical stimuli arises from the brainstem, the compensation of mechanical loads in awake humans is thought to involve suprapontine mechanisms. The aim of this study was to test this hypothesis by examining the effects of inspiratory resistive loading on the response of the diaphragm to transcranial magnetic stimulation.

**Results:**

Six healthy volunteers breathed room air without load (R0) and then against inspiratory resistances (5 and 20 cmH_2_O/L/s, R5 and R20). Ventilatory variables were recorded. Transcranial magnetic stimulation (TMS) was performed during early inspiration (I) or late expiration (E), giving rise to motor evoked potentials (MEPs) in the diaphragm (Di) and abductor pollicis brevis (APB). Breathing frequency significantly decreased during R20 without any other change. Resistive breathing had no effect on the amplitude of Di MEPs, but shortened their latency (R20: -0.903 ms, p = 0.03) when TMS was superimposed on inspiration. There was no change in APB MEPs.

**Conclusion:**

Inspiratory resistive breathing facilitates the diaphragm response to TMS while it does not increase the automatic drive to breathe. We interpret these findings as a neurophysiological substratum of the suprapontine nature of inspiratory load compensation in awake humans.

## Background

Breathing in humans fulfils both metabolic and behavioral functions. The automatic activity of brainstem central pattern generators continuously adapts ventilation to the production of carbon dioxide by the tissues. Suprapontine descending pathways convey voluntary and emotional respiratory commands that are independent of bodily requirements. Spinal respiratory motoneurons integrate the corresponding bulbospinal and corticospinal inputs [1,2], hence a reciprocal modulation of the two types of command. In one direction, direct corticospinal projections [3] account for voluntary disruptions of the respiratory rhythm during voluntary respiratory maneuvers or non-respiratory uses of the respiratory system (e.g. speech). In the other direction, increases in the automatic drive to breathe facilitate the diaphragm response to transcranial magnetic stimulation (TMS). This is true during quiet tidal breathing [4], where such inspiration-related facilitation has been described during wake and sleep. This is also true during CO2-stimulated breathing [5], that accelerates the diaphragm response to TMS applied during both inspiration and expiration.

The ventilatory responses to chemical stimuli such as hypoxia or hypercapnia are automatic and brainstem-generated. In contrast, the ventilatory adaptations to inspiratory mechanical loading in humans are believed to involve suprapontine mechanisms (see reviews in [6,7]). This postulate is generally called on to explain why awake humans faced with mechanical loads tend not to hypoventilate, whereas hypoventilation does develop in animals and anesthetized humans under similar conditions. The precise neural origin and pathway of inspiratory load compensation are not known. It could involve reflex mechanisms increasing the automatic drive to breathe, but there are arguments against this hypothesis in the literature [8-10]. It could also involve either or both of the known corticospinal pathways to the diaphragm (one from the primary motor cortex [11,12], the other from premotor areas [13,14]). In the latter case, resistive loading is not expected to increase the automatic drive to breathe but the response of the diaphragm to transcranial magnetic stimulation should be facilitated, because of spinal integration of the suprapontine command activated for load compensation. We designed the present study to test this hypothesis.

## Results

**Figure 1:**
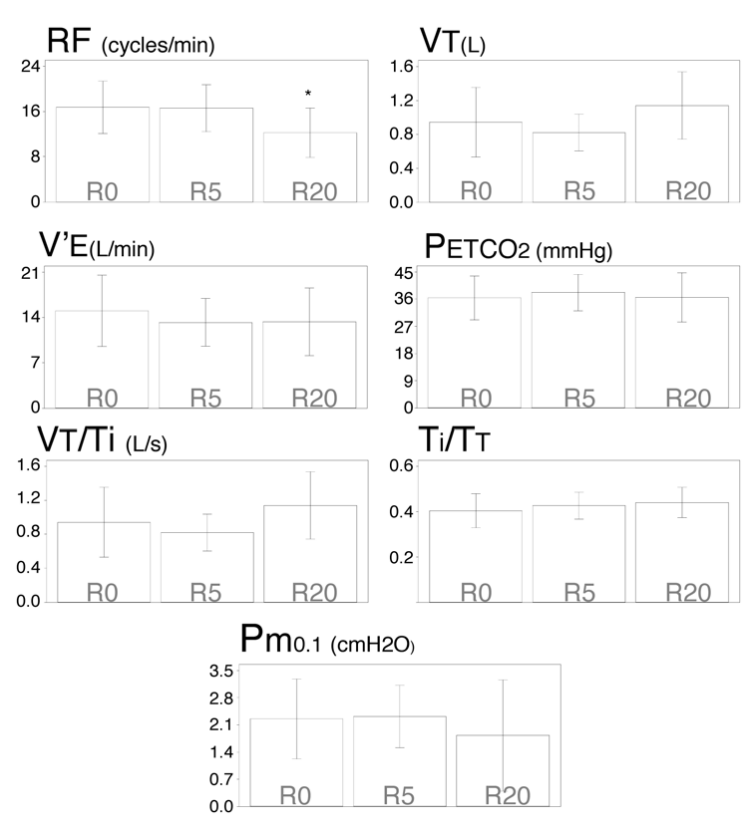
**Inspiratory resistive loading and breathing pattern**. Effectof breathing against inspiratory linear resistive loads onrespiratory frequency (RF), tidal volume (VT), minute ventilation(V'E), end-tidal carbon dioxide in the expired gas (PETCO2
 MathType@MTEF@5@5@+=feaafiart1ev1aaatCvAUfKttLearuWrP9MDH5MBPbIqV92AaeXatLxBI9gBaebbnrfifHhDYfgasaacH8akY=wiFfYdH8Gipec8Eeeu0xXdbba9frFj0=OqFfea0dXdd9vqai=hGuQ8kuc9pgc9s8qqaq=dirpe0xb9q8qiLsFr0=vr0=vr0dc8meaabaqaciaacaGaaeqabaqabeGadaaakeaacqqGqbaucqqGfbqrcqqGubavdaWgaaWcbaGaee4qamKaee4ta80aaSbaaWqaaiabikdaYaqabaaaleqaaaaa@339B@), mean inspiratory flow (VT/TI), duty cycle (TI/TT) and occlusion pressure (Pm_0.1_). Each bar corresponds to the mean of the corresponding data, with indication of ± 1 SD. The "*" stands for a significant difference at the 5% threshold.

Breathing through a 20 cmH_2_O/L/s inspiratory resistance (R20 condition) significantly reduced respiratory frequency as compared with unloaded breathing (R0 condition)(16.58 ± 1.37 cycles/min *versus *12.23 ± 0.83 cycles/min; p = 0.03). A 5 cmH_2_O/L/s inspiratory resistance (R5 condition) did not significantly affect respiratory frequency. There were slight increases in tidal volume that did not reach statistical significance; thus minute ventilation remained grossly unchanged. The end-tidal partial pressure in carbon dioxide in the expired gas (PETCO2
 MathType@MTEF@5@5@+=feaafiart1ev1aaatCvAUfKttLearuWrP9MDH5MBPbIqV92AaeXatLxBI9gBaebbnrfifHhDYfgasaacH8akY=wiFfYdH8Gipec8Eeeu0xXdbba9frFj0=OqFfea0dXdd9vqai=hGuQ8kuc9pgc9s8qqaq=dirpe0xb9q8qiLsFr0=vr0=vr0dc8meaabaqaciaacaGaaeqabaqabeGadaaakeaacqqGqbaucqqGfbqrcqqGubavdaWgaaWcbaGaee4qamKaee4ta80aaSbaaWqaaiabikdaYaqabaaaleqaaaaa@339B@) was unaffected by inspiratory resistances. This was also the case for the tidal volume to inspiratory time ratio (VT/TI), the inspiratory duty cycle or inspiratory to total time ratio (TI/TT), and one millisecond mouth occlusion pressure (Pm_0.1_)(Figure 1).

**Figure 2:**
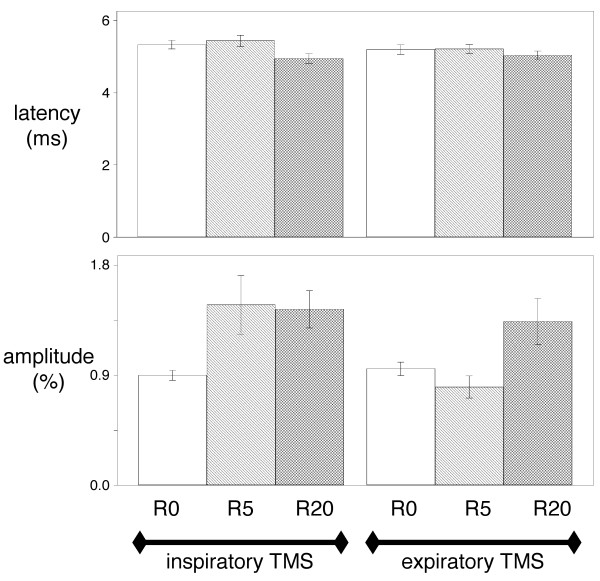
**Effects of inspiratory loading on the diaphragm EMG response to cervical magnetic stimulation**. Effect of breathing against inspiratory linear resistive loads on the latency (top) and the amplitude (bottom) of the electromyographic responses of the diaphragm to cervical magnetic stimulations (CMS) delivered during inspiration (left) or expiration (right). R0, R5 and R20 correspond to breathing against no resistance, against a resistance of 5 cmH_2_O/L/s, and against a resistance of 20 cmH_2_O/L/s, respectively. Vertical bars correspond to mean values with indication of ± 1 SD. Statistical analysis did not detect any significant difference at the 5% threshold.

Inspiratory resistive loading did not influence the esophageal pressure (Pes), gastric pressure (Pga) and transdiaphragmatic pressure (Pdi) to phenic nerve stimulation (cervical magnetic stimulation, CMS) during expiration or inspiration (Table 1), nor did it influence the corresponding latencies and amplitudes (Figure 2).

**Table 1:** Pressure responses to cervical magnetic stimulation

	**Cervical Magnetic Stimulation**					
	
	**R0**		**R5**		**R20**	
	
	Inspiration	Expiration	Inspiration	Expiration	Inspiration	Expiration
Pes	16.5 ± 11.9	12.1 ± 9.8	15.6 ± 8.6	11.8 ± 6.9	19.4 ± 17.2	9.2 ± 7.0
Pga	3.4 ± 3.6	3.7 ± 4.9	2.6 ± 1.6	2.7 ± 1.6	2.4 ± 1.4	2.7 ± 1.2
Pdi	19.9 ± 13.5	19.9 ± 10.9	18.2 ± 9.3	14.9 ± 7.0	21.8 ± 17.0	11.9 ± 7.5

Mean values (± SD) of esophageal (Pes), gastric (Pga) and transdiaphragmatic (Pdi) pressure (in cmH2O) in response to cervical magnetic stimulation while the subjects were breathing room air (R0) or against a resistance of either 5 (R5) or 20 (R20) cmH2O/l/s. Stimulations were applied either during early inspiration or late expiration.

**Figure 3:**
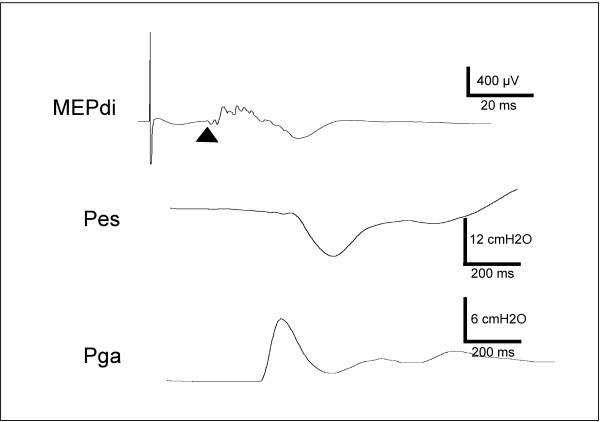
**Diaphragm response to transcranial magnetic stimulation delivered during loaded inspiration**. Example of the response of the diaphragm electromyogram (MEPdi, top), esophageal pressure (Pes, middle), and gastric pressure (Pga, bottom) to transcranial magnetic stimulation applied during inspiration in a subject breathing against a 5 cmH_2_0/L/s resistance. On the EMG trace, the triangle indicates the point at which the latency of the motor evoked potential (MEP) is measured.

**Figure 4:**
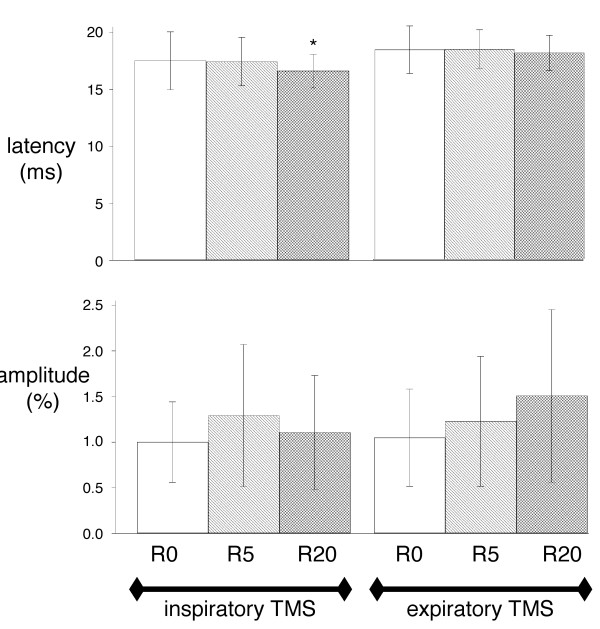
**Effects of inspiratory loading on the diaphragm EMG response to transcranial magnetic stimulation**. Effect of breathing against inspiratory linear resistive loads on the latency (top) and the amplitude (bottom) of the electromyographic responses of the diaphragm to transcranial magnetic stimulations (TMS) delivered during inspiration (left) or expiration (right). R0, R5 and R20 correspond to breathing against no resistance, against a resistance of 5 cmH_2_O/L/s, and against a resistance of 20 cmH_2_O/L/s, respectively. Vertical bars correspond to mean values with indication of ± 1 SD. The "*" stands for a significant difference at the 5% threshold.

The Pes, Pga and Pdi responses to transcranial magnetic stimulation (TMS)(Figure 3) were also unaffected by inspiratory resistive loading, both during inspiration and expiration (Table 2). There was no effect of inspiratory resistive loading on the amplitudes of the diaphragmatic motor evoked potentials (MEP) (Figure 4). Conversely, the latencies of the diaphragm MEPs obtained in response to inspiratory TMS were significantly shorter in the R20 condition than in the R0 condition (mean difference -0.903 ms, 95%CI from -1.79 to -0.016, p = 0.03) (Figure 4). This shortening was not observed in response to expiratory TMS.

**Table 2:** Pressure responses to transcranial magnetic stimulation

	**Transcranial Magnetic Stimulation**					
	
	**R0**		**R5**		**R20**	
	
	Inspiration	Expiration	Inspiration	Expiration	Inspiration	Expiration
Pes	5.8 ± 5.6	3.9 ± 5.8	7.1 ± 4.8	4.1 ± 6.3	8.7 ± 7.5	4.4 ± 4.0
Pga	1.6 ± 1.4	1.1 ± 2.0	1.6 ± 1.4	1.5 ± 2.6	1.3 ± 1.8	1.4 ± 1.7
Pdi	7.1 ± 6.8	5.1 ± 7.8	8.7 ± 6.1	5.6 ± 8.8	10.1 ± 8.5	5.8 ± 5.4

Mean values (± SD) of esophageal (Pes), gastric (Pga) and transdiaphragmatic (Pdi) pressure (in cmH2O) in response to transcranial magnetic stimulation while the subjects were breathing room air (R0) or against a resistance of either 5 (R5) or 20 (R20) cmH2O/l/s. Stimulations were applied either during early inspiration or late expiration.

**Figure 5:**
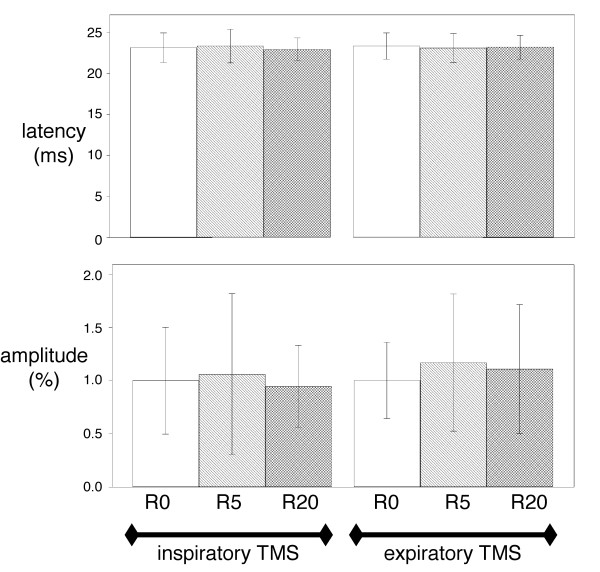
**Effects of inspiratory loading on the abductor pollicis brevis EMG response to transcranial magnetic stimulation**. Effect of breathing against inspiratory linear resistive loads on the latency (top) and the amplitude (bottom) of the electromyographic responses of the abductor pollicis brevis to magnetic transcranial stimulations (TMS) delivered during inspiration (left) or expiration (right). R0, R5 and R20 correspond to breathingagainst no resistance, against a resistance of 5 cmH_2_O/L/s, andagainst a resistance of 20 cmH_2_O/L/s, respectively. Vertical barscorrespond to mean values with indication of ± 1 SD. No significant differences were found between conditions.

The EMG responses of the abductor pollicis brevis to cervical and transcranial magnetic stimulations were not altered by inspiratory resistive breathing, both in terms of latency and amplitude (Figure 5).

## Discussion

**Figure 6:**
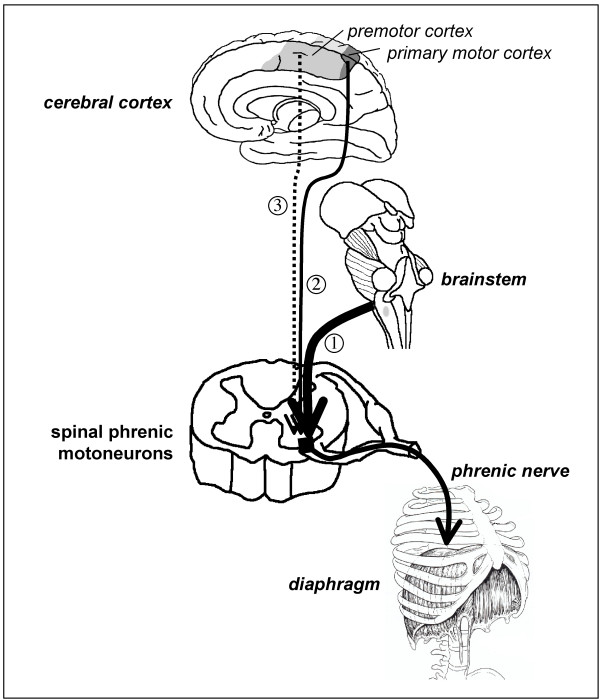
**Interactions between bulbospinal and corticospinal commands at spinal phrenic motoneuron level**. Schematic representation of the interactions between bulbospinal and corticospinal commands at the level of the spinal phrenic motoneuron. Arrow "1" represents the bulbospinal inputs to the spinal motoneurons, responsible for the production of ventilation and its automatic adaptation to bodily requirements. Arrow "2" represents the direct input from the primary motor cortex. This pathway carries voluntary respiratory commands, and is considered to be the main pathway activated by transcranial magnetic stimulation (TMS) as performed in this study. The diaphragm response to TMS depends on the polarization of the phrenic motoneurons when TMS is applied. It is therefore facilitated if TMS is superimposed on an increased bulbospinal drive (arrow 1) or on a voluntary contraction of the diaphragm (arrow 2). Arrow "3" represents the pathway from the premotor area of the cerebral cortex to the diaphragm [14]. Resistive inspiratory loading does not increase the bulbospinal drive but facilitates the response to TMS, possibly in line with the activation of this latter pathway.

In our subjects, and in line with previous observations [8-10,15], inspiratory resistive breathing did not increase the automatic ventilatory drive (and if anything tended to decrease it). In contrast, inspiratory loading facilitated the response of the diaphragm to transcranial magnetic stimulation. This indicates that sources other than the brainstem respiratory generators were activated by resistive breathing and increased the inputs received by the phrenic motoneurons. For reasons that will be discussed below, increased afferent traffic is unlikely and therefore our observations point to the involvement of suprapontine structures. We believe that these results provide a neurophysiological substratum to the behavioral nature of inspiratory load compensation in awake humans, and could open new perspectives regarding the functional role of the premotor representation of the diaphragm [14]. This is summarized in Figure 6.

### Mechanisms of inspiratory resistive induced facilitation of the diaphragm response to transcranial magnetic stimulation

The facilitation of the response of a muscle to single pulse TMS can occur at the cortical or at the spinal level. Cortical facilitation typically increases the size of the motor evoked potentials without latency changes [16,17]. Spinal facilitation, in contrast, tends to shorten latency. This can occur through the recruitment of additional motoneurons ("spatial" facilitation) that, in line with the size principle of motoneuron recruitment, have increasingly fast conduction properties. In this case, there is a simultaneous decrease in latency and increase in amplitude (that can also be the expression of concurrent cortical facilitation). This is typically what happens when TMS is superimposed on a voluntary contraction [18,19], and it has been repeatedly verified for the diaphragm [3,12,20-22]. Spinal facilitation can also occur in the absence of motoneuron recruitment. Indeed, single pulse TMS produces successive descending volleys, early ones through the direct excitation of pyramidal tract neurons ("D-waves"), and later ones through the activation of cortical interneurons ("I-waves"). Normally, spinal cells require the temporal summation of D- and I-waves to fire [19,23,24]. If pre-conditioned they can respond to early I-waves or D-waves, as evidenced by studies of single motor unit potentials [18] ("temporal" facilitation). For the diaphragm, spinal facilitation of the temporal type has been invoked to explain the effects of changes in the bulbospinal drive to breathe on the response to TMS [4,5]. Mehiri et al. [4] have indeed observed that superimposing TMS upon inspiration rather than expiration shortened the latency of the diaphragm response by about 1 ms without modifying the amplitude of the diaphragm MEPs. This effect was magnified when afferent and cortical traffics were minimized by sleep. Straus et al [5] showed that similar changes were provoked by breathing 7% CO_2_.

In the present study, an inspiratory resistance of 20 cmH_2_O/L/s significantly shortened the latency of the diaphragmatic response to inspiratory TMS. This shortening was 0.9 ms on average (Figure 4), which corresponds to similar changes observed with other experimental designs [4,5]. We did not observe a significant change in amplitude, but we acknowledge that this could be due to insufficient statistical power (Figure 4). However, even if this was the case, the effect of resistive breathing on MEPs amplitudes would still be much less marked than the effect on latencies. According to the above described mechanisms and observations, we postulate that the facilitation of the diaphragm response to transcranial magnetic stimulation that we observed occurred at the spinal level. Of note, the lack of influence of resistive breathing on the response of the abductor pollicis brevis to transcranial magnetic stimulation indicates that the observed phenomenon was specific to the diaphragm rather than concerning the corticospinal tract globally. Whatever the source of the facilitation of the diaphragm response to TMS that inspiratory resistive breathing provoked (see below), this interpretation of our findings is coherent with the integration of afferent, segmental and supraspinal inputs that is characteristic of phrenic motoneurons [1,2].

### Putative source of facilitation during resistive breathing (Figure 6)

Conceivably, reflex mechanisms increasing the automatic drive to breathe could explain the compensation of inspiratory loading in humans, but data in the literature goes against this hypothesis [8-10]. Our subjects did not exhibit signs of increased ventilatory drive when confronted with resistive loading (no change in VT/TI or Pm_0.1_). On the contrary, they exhibited a decreased breathing frequency. The discrepancy between frequency and VT/TI or Pm_0.1 _probably illustrates the differences in the control of the intensity of ventilatory drive and that of its timing. As a result, it seems safe to rule out facilitation through increased bulbospinal inputs as the explanation of our observations. We acknowledge that, because stimulations were triggered at a fixed Pes level and because resistive breathing slowed respiration down, TMS was delivered later in inspiration during resistive breathing than during unloaded breathing. The corresponding interval was however sufficiently narrow (150–300 ms) not to influence the diaphragm MEPs latencies (unpublished observations by our group).

TMS was delivered at a fixed level of inspiratory pressure, and sufficiently early during inspiration for the peak activity of inspiratory muscles to have not yet occurred [6]. Therefore, the afferent traffic from the diaphragm was probably not very different during free breathing and during loaded breathing. This makes facilitation from afferent stimulation all the more unlikely as the costal diaphragm is poor in spindles [25]. In addition, afferent stimulation is associated with larger MEPs [26], which we did not observe.

The remaining explanation for the inspiratory resistive breathing related facilitation of the diaphragm response to TMS would be the activation of suprapontine regions. Indeed, fighting an inspiratory resistance can activate multiple such regions [7,27]. The primary motor representation of the diaphragm is not a good candidate because our subjects were not instructed to fight the load, and had to breathe against it in a sustained manner. Voluntary load compensation is thus not likely. It would have been expected to induce "spatial" facilitation with an increase in amplitude of diaphragm MEPs [12,28], which we failed to observe. Finally, inspiratory loading does not seem to activate the sensorimotor cortex [29]. Inspiratory muscles obey premotor commands [13] likely to originate in the medial premotor cortex [30]. There is a cortico-diaphragmatic pathway originating in the supplementary motor area [14]. However, the exact "respiratory role" of premotor regions is unknown. These regions are involved in the preparation of movement. Their activation could hypothetically be called upon to explain the progressive change in breathing pattern that follows the imposition of an inspiratory load, with a shift from an initially highly variable respiration to a much more steady one [6]. As there are direct projections of premotor areas to the phrenic spinal motoneurons, it is reasonable to postulate that the activation of the premotor control of the diaphragm would depolarize them and thus increase their "receptiveness" to a concurrent input (Figure 6), which would explain our findings. This hypothesis is supported by the demonstration that a premotor negativity has a facilitating effect on the response of the target muscle to TMS [31]. It must be emphasized that the facilitation of the diaphragm response that we observed is most certainly independent of any direct activation of the premotor diaphragm pathway that is best stimulated with a 110 mm double cone coil orientated antero-posteriorly and has a higher motor threshold than the primary motor diaphragm pathway [14].

## Conclusion

In summary, we submit that inspiratory load compensation in awake humans may involve the activation of a premotor cortical area. Confirmatory studies are necessary, but this information could be relevant to a better understanding of the respiratory sensations elicited by inspiratory loading, and possibly to the pathophysiology of diseases where inspiratory load compensation is mandatory to sustain ventilation. In this view, it has been shown that patients with chronic obstructive pulmonary disease (COPD) tend to exhibit facilitated diaphragm response to TMS [32]. Understanding whether, and in what proportion, this is due to an increased ventilatory drive, inspiratory loading, or both would probably be worthwhile.

## Methods

### Subjects

Six healthy subjects (2 women, 4 men, 22–25 years-old) participated in the study after ethical and legal clearance (Comité Consultatif de Protection des Personnes se prêtant à des Recherches Biomédicales Pitié-Salpêtière). They were naive to respiratory physiology experiments, were not sleep deprived, and had been instructed to refrain from consuming alcohol or psychotropic substances of any kind during the preceding 24 hours. They received detailed information and gave written consent.

### Ventilation and respiratory pressures

The subjects were studied seated, with abdomen unbound and wearing a nose clip. They breathed through a mouth piece connected to a pneumotachometer and a non-rebreathing two-way valve (Hans Rudolph, Kansas City, MO, USA) for the measurement of respiratory rhythm, tidal volume, minute ventilation, mean inspiratory flow (VT/TI) and duty cycle (TI/TT)(Respiratory Pressure Module, MedGraphics, Medical Graphic Corporation, Saint Paul, Minnesota, USA). PETCO2
 MathType@MTEF@5@5@+=feaafiart1ev1aaatCvAUfKttLearuWrP9MDH5MBPbIqV92AaeXatLxBI9gBaebbnrfifHhDYfgasaacH8akY=wiFfYdH8Gipec8Eeeu0xXdbba9frFj0=OqFfea0dXdd9vqai=hGuQ8kuc9pgc9s8qqaq=dirpe0xb9q8qiLsFr0=vr0=vr0dc8meaabaqaciaacaGaaeqabaqabeGadaaakeaacqqGqbaucqqGfbqrcqqGubavdaWgaaWcbaGaee4qamKaee4ta80aaSbaaWqaaiabikdaYaqabaaaleqaaaaa@339B@ was monitored in the expiratory gas with an infra-red gas analyzer (Medical gas Analyzer LB-2 Beckman, California, USA). Pm_0.1 _was measured at the mouth as follows. With the subjects breathing through a two-way valve separating the inspiratory and the expiratory limbs of the circuit, the inspiratory limb was occluded silently using an inflatable balloon during a randomly selected expiration. In this way, the next inspiration was performed against an occluded airway. The occlusion pressure was defined as the value reached 100 ms after the beginning of the ensuing drop in mouth pressure, namely at a time too early for the occlusion to have been perceived by the subject. The upper airway was freed by deflating the occlusion balloon within 3–400 ms of the beginning of effort. Pm_0.1 _measurements were performed every four to seven breathing cycles. The values hereafter provided are the average of at least ten steady-state measurements in each condition for each subject. Pes and Pga were measured using two air-filled (1 ml) balloon catheters inserted through the nose (length 80 cm, 1.5 mm internal diameter, Marquat, Boissy Saint Léger, France) and connected to linear differential pressure transducers (Validyne MP45, ± 100 cmH_2_O, Northridge, CA, USA).

### Electromyograms (EMG)

Surface recordings of the right diaphragmatic electromyogram were obtained using a pair of skin-taped silver cup electrodes filled with conductive paste and positioned on the chest according to a technique previously described as minimizing the risk of signal contamination by the activity of extradiaphragmatic muscles [33,34]. In brief, the active electrode was placed in the lowest accessible intercostal space, between the midclavicular line and the lateral edge of the sternum. The reference electrode was on the rib above, at a 2 cm distance. The surface electromyogram of a hand muscle, the right abductor pollicis brevis, was simultaneously recorded to serve as control. EMG signals were amplified, band pass filtered (20 Hz – 5 kHz) digitized (10 kHz) and stored as computer files for subsequent analysis (Neuropack Sigma, Nihon Kohden, Tokyo, Japan).

### Stimulations

All magnetic stimulations were carried out with a Magstim 200 stimulator equipped with a 90 mm circular coil and set to its maximum output (2.5 Tesla)(Magstim, Sheffield, UK). Cervical magnetic stimulation (CMS) [20] was used to describe the diaphragmatic responses to peripheral phrenic stimulation. Transcranial magnetic stimulation (TMS) was achieved with the coil placed over the vertex, after optimization of the response and careful maintenance of the coil position. CMS and TMS were performed during either late expiration or early inspiration. The stimulator was triggered from the Pes signal, with a threshold value that was always identical and set slightly below end-expiratory Pes. With this approach, expiratory stimulations occurred at a late phase of expiration, namely at a time where a residual post-inspiratory activity was most unlikely. The timing of inspiratory stimulations was thus not fixed by definition, and depended on the rate of change of Pes. Nevertheless, all the inspiratory stimulations occurred between 150 and 300 ms after the beginning of the Pes drop.

### Data analysis

Responses to stimulations were observed in terms of Pes and Pga (noted "*cms*" or "*tms*" depending on the site of stimulation) and of motor evoked potentials. MEP amplitudes were measured between the highest and the lowest peak of the evoked responses. MEP latencies were measured as the time elapsed between the stimulus and the first departure of the signal from baseline (Figure 3). Pes,*cms*, Pes,*tms*, Pga,*cms *and Pga,*tms *were calculated as the difference between the value at the time of stimulation and the peak of the ensuing pressure wave. The corresponding Pdi values were calculated off line by subtracting Pes from Pga. The values reported for each subject are the average of five CMS and ten TMS.

### Effect on breathing through linear inspiratory resistances

The subjects first accustomed themselves to the experimental setting by calmly breathing room air without added resistance (R0) through the apparatus. Ventilatory variables were measured and CMS and TMS were performed. Then inspiratory linear resistances of 5 cmH_2_O/L/s (R5) and 20 cmH_2_O/L/s (R20) (Hans Rudolph, 7100 R5 and R20, Kansas City, MO, USA) were added to the circuit in random order. Ten minutes were allowed for stabilization, after which ventilatory variables were again measured before the application of CMS and TMS. The subjects did not receive any instructions on how to behave when confronted with inspiratory loading.

### Statistical analysis

It was conducted using Statistix^® ^software (v 8.0, Tallahassee, FL, USA). The normality of data distributions as tested with the Shapiro-Wilk test being consistently confirmed, the results were described in terms of means ± SD and linear models were used. The ventilatory variables and respiratory pressures taken as dependent variables were submitted to an analysis of variance for repeated measures (with the "subject" variable as a random factor, and the loading condition as the within-subjects factor). The responses to TMS were studied with a nested split-plot design, to separately analyze the effects of inspiratory loading and stimulation timing. Dependent variables were the Pdi responses to TMS and the amplitudes and latencies of the diaphragm and the abductor pollicis brevis MEPs. The stimulation timing factors were randomly assigned to the subjects (main plot) and the loading condition factors were assigned to the split plot. Post-hoc comparisons were conducted in reference to the R0 condition using Dunnett's procedure. The results were considered significant when the probability p of a type I error was less than 5%.

## Authors' contributions

CL participated in study design, carried out data acquisition, analyzed the results, and helped to draft the manuscript; MR participated study design, contributed significantly (or greatly) to data interpretation, and contributed to the writing of the manuscript; MNF contributed to data interpretation and to the writing of the manuscript; CMP contributed to data analysis and interpretation and to the writing of the final manuscript; MZ and JPD critically reviewed the design of the study and the manuscript, with important impact on intellectual content; TS and CS were responsible for study design and statistical processing of data; they contributed the largest part of data interpretation and produced the final manuscript that received approval from all authors.

## References

[B1] Aminoff MJ, Sears TA (1971). Spinal integration of segmental, cortical and breathing inputs to thoracic respiratory motoneurones. J Physiol.

[B2] Sears TA, Aminoff MJ (1970). Spinal integration of cortical, brainstem, and segmental inputs to thoracic respiratory motoneurons. Neurology.

[B3] Corfield DR, Murphy K, Guz A (1998). Does the motor cortical control of the diaphragm 'bypass' the brain stem respiratory centres in man?. Respir Physiol.

[B4] Mehiri S, Straus C, Arnulf I, Attali V, Zelter M, Derenne JP, Similowski T (2006). Responses of the diaphragm to transcranial magnetic stimulation during wake and sleep in humans. Respir Physiol Neurobiol.

[B5] Straus C, Locher C, Zelter M, Derenne JP, Similowski T (2004). Facilitation of the diaphragm response to transcranial magnetic stimulation by increases in human respiratory drive. J Appl Physiol.

[B6] Younes M, Dempsey JA (1995). Mechanisms of respiratory load compensation. Regulation of breathing.

[B7] Horn EM, Waldrop TG (1998). Suprapontine control of respiration. Respir Physiol.

[B8] Clague JE, Carter J, Pearson MG, Calverley PM (1992). Effort sensation, chemoresponsiveness, and breathing pattern during inspiratory resistive loading. J Appl Physiol.

[B9] Lopata M, Onal E, Ginzburg AS (1983). Respiratory muscle function during CO2 rebreathing with inspiratory flow-resistive loading. J Appl Physiol.

[B10] Ramonatxo M, Mercier J, Cohendy R, Prefaut C (1991). Effect of resistive loads on pattern of respiratory muscle recruitment during exercise. J Appl Physiol.

[B11] Gandevia SC, Rothwell JC (1987). Activation of the human diaphragm from the motor cortex. J Physiol (Lond).

[B12] Similowski T, Straus C, Coic L, Derenne JP (1996). Facilitation-independent response of the diaphragm to cortical magnetic stimulation. Am J Respir Crit Care Med.

[B13] Macefield G, Gandevia SC (1991). The cortical drive to human respiratory muscles in the awake state assessed by premotor cerebral potentials. J Physiol.

[B14] Sharshar T, Hopkinson NS, Jonville S, Prigent H, Carlier R, Dayer MJ, Swallow EB, Lofaso F, Moxham J, Polkey MI (2004). Demonstration of a second rapidly conducting cortico-diaphragmatic pathway in humans. J Physiol.

[B15] Lopata M, La Fata J, Evanich MJ, Lourenco RV (1977). Effects of flow-resistive loading on mouth occlusion pressure during CO2 rebreathing. Am Rev Respir Dis.

[B16] Kasai T, Kawai S, Kawanishi M, Yahagi S (1997). Evidence for facilitation of motor evoked potentials (MEPs) induced by motor imagery. Brain Res.

[B17] Kiers L, Fernando B, Tomkins D (1997). Facilitatory effect of thinking about movement on magnetic motor-evoked potentials. Electroencephalogr Clin Neurophysiol.

[B18] Calancie B, Nordin M, Wallin U, Hagbarth KE (1987). Motor-unit responses in human wrist flexor and extensor muscles to transcranial cortical stimuli. J Neurophysiol.

[B19] Thompson PD, Day BL, Rothwell JC, Dressler D, Maertens de Noordhout A, Marsden CD (1991). Further observations on the facilitation of muscle responses to cortical stimulation by voluntary contraction. Electroencephalogr Clin Neurophysiol.

[B20] Similowski T, Fleury B, Launois S, Cathala HP, Bouche P, Derenne JP (1989). Cervical magnetic stimulation: a new painless method for bilateral phrenic nerve stimulation in conscious humans. J Appl Physiol.

[B21] Maskill D, Murphy K, Mier A, Owen M, Guz A (1991). Motor cortical representation of the diaphragm in man. J Physiol (Lond).

[B22] Davey NJ, Murphy K, Maskill DW, Guz A, Ellaway PH (1996). Site of facilitation of diaphragm EMG to corticospinal stimulation during inspiration. Respir Physiol.

[B23] Hess CW, Mills KR, Murray NM (1987). Responses in small hand muscles from magnetic stimulation of the human brain. J Physiol.

[B24] Kaneko K, Kawai S, Fuchigami Y, Shiraishi G, Ito T (1996). Spinal cord potentials after transcranial magnetic stimulation during muscle contraction. Muscle Nerve.

[B25] Duron B, Hornbein TF (1981). Intercostal and diaphragmatic muscle endings and afferents. Regulation of Breathing, part I.

[B26] Komori T, Watson BV, Brown WF (1992). Influence of peripheral afferents on cortical and spinal motoneuron excitability. Muscle Nerve.

[B27] Fink GR, Corfield DR, Murphy K, Kobayashi I, Dettmers C, Adams L, Frackowiak RS, Guz A (1996). Human cerebral activity with increasing inspiratory force: a study using positron emission tomography. J Appl Physiol.

[B28] Sharshar T, Ross E, Hopkinson NS, Dayer M, Nickol A, Lofaso F, Moxham J, Similowski T, Polkey MI (2003). Effect of voluntary facilitation on the diaphragmatic response to transcranial magnetic stimulation. J Appl Physiol.

[B29] Isaev G, Murphy K, Guz A, Adams L (2002). Areas of the brain concerned with ventilatory load compensation in awake man. J Physiol.

[B30] Ball T, Schreiber A, Feige B, Wagner M, Lucking CH, Kristeva-Feige R (1999). The role of higher-order motor areas in voluntary movement as revealed by high-resolution EEG and fMRI. Neuroimage.

[B31] Zaaroor M, Pratt H, Starr A (2003). Time course of motor excitability before and after a task-related movement. Neurophysiol Clin.

[B32] Hopkinson NS, Sharshar T, Ross ET, Nickol AH, Dayer MJ, Porcher R, Jonville S, Moxham J, Polkey MI (2004). Corticospinal control of respiratory muscles in chronic obstructive pulmonary disease. Respir Physiol Neurobiol.

[B33] Demoule A, Verin E, Locher C, Derenne JP, Similowski T (2003). Validation of surface recordings of the diaphragm response to transcranial magnetic stimulation in humans. J Appl Physiol.

[B34] Verin E, Straus C, Demoule A, Mialon P, Derenne JP, Similowski T (2002). Validation of improved recording site to measure phrenic conduction from surface electrodes in humans. J Appl Physiol.

